# Leukocytes, Systemic Inflammation and Immunopathology in Acute-on-Chronic Liver Failure

**DOI:** 10.3390/cells9122632

**Published:** 2020-12-08

**Authors:** Mireia Casulleras, Ingrid W. Zhang, Cristina López-Vicario, Joan Clària

**Affiliations:** 1 Biochemistry and Molecular Genetics Service, Hospital Clínic-IDIBAPS, 08036 Barcelona, Spain; mcasulleras@clinic.cat (M.C.); iwzhang@clinic.cat (I.W.Z.); 2 European Foundation for the Study of Chronic Liver Failure (EF Clif) and Grifols Chair, 08021 Barcelona, Spain; 3 Department of Biomedical Sciences, School of Medicine and Health Sciences, Universitat de Barcelona, 08036 Barcelona, Spain

**Keywords:** advanced liver disease, systemic inflammation, immunosuppression, immunometabolism, mononuclear phagocytes, cytokines, bioactive lipid mediators

## Abstract

Acute-on-chronic liver failure (ACLF) is a complex syndrome that develops in patients with cirrhosis and is characterized by acute decompensation, organ failure(s) and high short-term mortality. ACLF frequently occurs in close temporal relationship to a precipitating event, such as acute alcoholic, drug-induced or viral hepatitis or bacterial infection and, in cases without precipitating events, probably related to intestinal translocation of bacterial products. Dysbalanced immune function is central to its pathogenesis and outcome with an initial excessive systemic inflammatory response that drives organ failure and mortality. This hyperinflammatory state ultimately impairs the host defensive mechanisms of immune cells, rendering ACLF patients immunocompromised and more vulnerable to secondary infections, and therefore to higher organ dysfunction and mortality. In this review, we describe the prevailing characteristics of the hyperinflammatory state in patients with acutely decompensated cirrhosis developing ACLF, with special emphasis on cells of the innate immune system (i.e., monocytes and neutrophils), their triggers (pathogen- and damage-associated molecular patterns [PAMPs and DAMPs]), their effector molecules (cytokines, chemokines, growth factors and bioactive lipid mediators) and the consequences on tissue immunopathology. In addition, this review includes a chapter discussing new emerging therapies based on the modulation of leukocyte function by the administration of pleiotropic proteins such as albumin, Toll-like receptor 4 antagonists, interleukin-22 or stem cell therapy. Finally, the importance of finding an appropriate intervention that reduces inflammation without inducing immunosuppression is highlighted as one of the main therapeutic challenges in cirrhosis.

## 1. Acute-on-Chronic Liver Failure (ACLF) 

ACLF is a severe syndrome evolving in patients with acutely decompensated (AD) liver cirrhosis. ACLF is characterized by the manifestation of organ dysfunctions and failures across the six major organ systems (liver, kidney, brain, coagulation, circulation, and respiration) resulting in high short-term mortality (28-day mortality of 32%) [1,2,3]. The liver and the kidney are the most commonly affected organ systems followed by coagulation, brain, circulation and respiration. ACLF is classified in three grades of severity (ACLF-1, -2 and -3) according to the number of organ failures and may exhibit a variable course during hospitalization as it can follow a steady course or resolve, improve or worsen within a few days. The CANONIC study, a prospective observational investigation in 1343 patients hospitalized for acute decompensation of cirrhosis, provided the first evidence-based definition of ACLF which includes the presence of organ failure(s) and a 28-day mortality risk of 15% or higher [1]. In Western countries, ACLF is particularly prevalent among young patients with alcoholic cirrhosis and in 60% of the cases develops in close association with potential precipitating events, mainly bacterial infections or active alcoholism. In Asian countries, ACLF is more commonly diagnosed in patients with hepatitis B-related cirrhosis who exhibit lower prevalence of extrahepatic organ failures. 

## 2. Systemic Inflammation and Immunopathology Are Major Drivers of ACLF

Recent advances in our understanding of the pathophysiological basis of ACLF indicate that a systemic hyperinflammatory state is the main driver of widespread tissue and organ injury in patients with AD cirrhosis developing ACLF [4,5]. This hyperinflammatory state is produced by the massive release of inflammatory mediators such as cytokines, chemokines, growth factors and bioactive lipid mediators (see below) that lead to immune-mediated tissue damage, a process that is also known as immunopathology. For example, in the microvasculature of vital organs, proinflammatory cytokines damage the endothelium glycocalyx and trigger neutrophil and monocyte adhesion to endothelial cells and their transmigration into tissues [6]. Activated immune cells, in turn, release mediators such as proteases, oxidative molecules, cytotoxic cytokines, prostaglandins (PGs) and leukotrienes (LTs) (see below), which further intensify tissue damage. 

At present little is known about the identity of the triggers (either of infectious or noninfectious origin) leading to immune cell activation and immunopathology in patients with AD cirrhosis evolving to ACLF. Bacterial infections are present in 33% of cases of ACLF and therefore pathogen-associated molecular patterns (PAMPs) released by infecting bacteria are likely contributing [7]. In addition, circulating PAMPs can be the result of the translocation of bacterial products from the intestinal lumen to the systemic circulation. In fact, bacterial overgrowth, increased permeability of the intestinal mucosa, and impaired function of the intestinal innate immune system are common in AD patients developing ACLF [8,9]. PAMPs are unique conserved molecular structures that are recognized by the host via dedicated receptors called pattern-recognition receptors (PRRs), including among others Toll-like receptors (TLRs) present at the cell surface or in the endosomal compartment and NOD-like receptors (NLRs), present in the cytosol of the cells [10]. These receptors recognize nucleic acids and protein, lipid and carbohydrate components characteristic of bacteria and viruses. The engagement of PRRs results in the stimulation of signaling cascades that activate transcription factors such as nuclear factor (NF)-kB or activator protein 1 [11], which in turn induce the expression of a battery of genes encoding for molecules involved in inflammation (i.e., interleukin 6 (*IL6*) and tumor necrosis factor α (*TNF*). Lipopolysaccharide (LPS) from the cell wall of Gram-negative bacteria, which engages TLR4-mediated activation of multiple downstream signaling pathways that result in the synthesis of cytokines and interferons, is a prime example of PAMPs [11]. 

Systemic inflammation can occur in patients with AD cirrhosis and ACLF in the absence of bacterial infections and/or bacterial translocation as the result of the release of damage-associated molecular patterns (DAMPs) from injured organs and tissues. DAMPs are released by dead, dying or injured cells and originate from several cellular compartments, especially from the nucleus (high mobility group box 1 (HMGB1) and histones), mitochondria (mitochondrial DNA and formyl peptides) and the cytosol (adenosine triphosphate (ATP)) [10]. Apart from necrosis, other immunogenic forms of cell-death such as necroptosis and pyroptosis are common in advanced liver disease and contribute to the enhanced release of DAMPs in this condition [12]. Similar to PAMPs, DAMPs initiate an immune response by binding to specific PRRs. In certain cases, inflammatory cytokines such as IL-1α and IL-33 can act as DAMPs and trigger inflammation through binding to their respective MyD88-coupled cognate receptors.

The intensity of systemic inflammation and the response of the immune system to PAMPs and DAMPs may depend on host genetic factors. For example, single-nucleotide variants might modulate the release of inflammatory molecules by innate immune cells or might induce changes in the expression of PRRs, such as TLRs. Consistent with this, genetic variants in genes coding for receptors of the innate immune system such as nucleotide-binding oligomerization domain 2 (NOD2) or ligands as mannan-binding lectin (MBL) and MBL-associated serine proteases (MASP) 2 have been shown to associate with increased short-term mortality in AD and ACLF patients [13]. Moreover, single nucleotide polymorphisms within the IL-1 gene cluster have been reported to protect patients with AD cirrhosis from uncontrolled systemic inflammation and to reduce the predisposition of these patients to develop ACLF [14]. 

## 3. Immunosuppression Is a Common Feature in ACLF

The hyperinflammatory response in patients with ACLF frequently occurs in parallel with the presence of dysfunctional innate immune system at the humoral, physical and cell-mediated level [8,15,16]. Due to hepatocellular insufficiency, cirrhotic patients commonly display reduced humoral anti-defense capacities as a result of decreased production of acute phase proteins, hypoalbuminemia and defective complement system [17,18,19]. Additionally, the physical barrier in cirrhosis is impaired, and even more so in ACLF, with gut leakage and dysfunction of the vasculature and sinusoidal endothelium being the most prominent features. 

Taking all these components of the innate immune system into account, the overall immune status in patients with ACLF ranges in the spectrum from immunosuppressive/immunoregulatory/tolerogenic to exuberantly hyperinflammatory, and these extremes are not mutually exclusive. Rather the contrary, these two conditions frequently coexist in the same patient, as a constant and persistent hyperinflammatory milieu characterized by increased circulating proinflammatory and anti-inflammatory mediators (e.g., galectin-3, IL-6, TNFα, IL-10) [4,20] and lipid mediators such as PGE_2_ can cause the downplaying of innate immune defensive responses and the expansion of regulatory immune cells leading to immunosuppression [21,22], probably in an attempt to keep the proinflammatory response at bay. The predominance of one or the other depends on temporal and spatial aspects: immune cells in the circulation may behave differently (inflammatory phenotype with production of cytokines) than their counterparts in the liver for example (more tolerogenic phenotype), and the immunodeficient phenotype tends to assume greater importance with increasing disease severity [23]. 

Bernsmeier et al. proposed a model which harmonizes these two extremes: Circulating inflammatory mediators, released by immune cells in an excessive manner in cirrhosis in response to PAMPs and DAMPs induce the formation of immunoregulatory monocytes/macrophages [20]. Thereupon, these cells migrate across endothelia facilitated by endothelial dysfunction into inflamed tissues. In ACLF, tissue macrophages tend to display functionally endotoxin tolerant/immunoregulatory features. Due to their enhanced migratory potential, these cells reverse migrate into the circulation, where they further expand into other tissues and lymph nodes, contributing to the immunosuppressive phenotype in ACLF [20]. The clinical consequence is increased susceptibility to bacterial infections as major precipitating event of organ failure(s), which is the discriminant feature of ACLF [1]. 

## 4. Portal Hypertension, Endothelial Activation and the Interplay with the Innate Immune System

The PREDICT study, a European-wide prospective study, recently identified portal hypertension as the second major pathophysiological mechanism in ACLF [24]. It is well-recognized that increased shunting in the context of portal hypertension leads to insufficient clearance of bacterial products and escape of bacteria from the reticuloendothelial system [25]. Therefore, innate immune activation is intricately linked to portal hypertension, as the latter favors bacterial translocation. For instance, bacterial translocation leads to stimulation of TLR4-mediated signaling in hepatic stellate cells, Kupffer cells, and liver sinusoidal endothelial cells and to a pre-activation of the innate immune system facilitating an exaggerated inflammatory response [26,27]. Interestingly, systemic inflammation can be dampened by reduction of the portal pressure via insertion of a transjugular intrahepatic portosystemic shunt, although systemic inflammation often still persists thereafter [28,29].

The interplay between the immune system and endothelial activation is illustrated by the fact that the inflammatory microenvironment favors the formation of microthrombi in the microvasculature of different organs [30] and targets the endothelium to release procoagulant factors [31,32], thereby contributing to immunopathology. Indirect markers of endothelial activation such as angiopoietin 2 [33], which recruits inflammatory cells and promotes cytokine-induced vascular leakage, and vascular cell adhesion molecule 1 (VCAM-1) [34] are markedly elevated in the circulation of patients with AD cirrhosis and ACLF. VCAM-1 participates in the adhesion of leukocytes to the endothelium and trans-endothelial migration. It is associated with multiorgan failure and in-hospital mortality in patients with severe sepsis [35]. Moreover, microparticles of leuko-endothelial, lymphocyte and hepatocyte origin, whose release is stimulated by LPS [36] are increased in plasma of patients with cirrhosis and correlate with their severity [37].

## 5. Immunometabolism Also Plays a Critical Role in ACLF 

Under normal conditions, mammalian cells obtain the vast majority of energy from mitochondrial oxidative phosphorylation (OXPHOS), which combines electron transport with cell respiration and ATP synthesis [38]. However, upon inflammatory conditions, mitochondria become dysfunctional and cells shift from producing ATP by OXPHOS to aerobic glycolysis [38,39,40]. Aerobic glycolysis (also known as Warburg effect) ultimately produces lactate and generates 2 ATP molecules per glucose molecule, thus is less efficient than OXPHOS, which generates about 36 ATPs per each molecule of glucose [38,39,40]. An important aspect to consider is that lactate as end-product of glycolysis is secreted in high amounts by innate immune cells upon activation, and that this metabolite acts as a negative feedback to limit inflammation by decreasing cytokine production and migration of monocytes and macrophages [41,42]. Nevertheless, a clear disadvantage of glycolysis is that it highly depends on glucose as a sole fuel source, whereas mitochondrial OXPHOS has more metabolic flexibility and can use fatty acids and amino acids for example as carbon sources [40]. Patients with AD cirrhosis also exhibit increased blood levels of intermediates of the pentose phosphate pathway, which branches off from glycolysis at the first committed step of glucose metabolism, suggesting that cytosolic glucose metabolism through alternative routes to glycolysis is also common in these patients [43]. At early stages of injury, energy homeostasis is still attainable through diverting energy production from OXPHOS to glycolysis. However, this is a short-term solution because cells are unable to sustain high energy production from glycolysis at more advanced stages of chronic liver disease [44]. Furthermore, impaired OXPHOS is also linked to an enhanced production of reactive oxygen species (ROS) [45]. 

The prevailing metabolic alteration in patients with AD cirrhosis and ACLF is also characterized by intense proteolysis and lipolysis releasing high amounts of amino acids and fatty acids, respectively, as well as by severe amino acid catabolism [43,46]. Similar catabolic processes are observed in other critical illnesses associated with systemic inflammation and multiorgan failure, such as sepsis or trauma [47], pointing to the systemic hyperinflammatory state present in patients with AD cirrhosis as the origin of their metabolic alteration, although the contribution of microbiota and metabolites of microbial origin cannot be excluded [43,48]. Nevertheless, the finding that the higher the plasma levels of inflammatory markers, the higher the intensity of the metabolic alteration positions systemic inflammation as its major driving force [43]. The goal of the intense catabolic metabolism in patients with AD cirrhosis is to provide nutrients to the energetically expensive inflammatory response, which must face with the production of inflammatory mediators, immune cell proliferation and migration, respiratory burst, and production of acute-phase proteins [47]. This energetically expensive systemic inflammatory response requires reallocation of stored nutrients to fuel immune activation. To cope with this, immune cells compete for energy with other maintenance programs, including those ensuring proper functioning of peripheral organs. Ultimately, the energetic trade-off between immune activation and organ function homeostasis may lead to peripheral organ hypometabolism and organ dysfunction and failure in these patients. 

## 6. Cells of the Innate Immune System: Role in ACLF

Ongoing studies are currently attempting to decipher the relative role of the innate versus the adaptive immune system in the pathogenesis of systemic inflammation in patients with AD cirrhosis evolving to ACLF. Although this dichotomy has not been resolved yet, plasma levels of cytokines involved in reshaping the adaptive immune system (i.e., IFN-γ, IFN-α2 and IL-17A) were not statistically different in patients with ACLF and patients with AD cirrhosis [4], suggesting that, similar to sepsis [49], the innate immune system plays a major contributory role to ACLF development. Consistent with this view, ACLF patients display increased leukocyte count compared to those with AD cirrhosis [1], in particular increased neutrophil and monocyte counts but accompanied by lymphopenia [50]. Information about other cell types of the innate immune system such as dendritic cells (DCs) and natural killer cells (NKs) is scarce, but decreased number of NK cells and attenuated function (i.e., cytotoxicity and killing activity) have been shown to predispose patients with HBV-related ACLF to infection [51]. Because of their predominance in the circulation and their primary role in protecting cirrhotic patients against secondary infections [52,53], in the following paragraphs we will focus our discussion on monocytes and neutrophils. 

### 6.1. Mononuclear Phagocytes

Cirrhosis is commonly associated with the presence of monocytosis [54]. In 2005, before the existence of a widely accepted definition of ACLF, Wasmuth et al. had already described impaired antigen-presentation capacities and functional deactivation of monocytes from patients with AD liver cirrhosis [55]. In contrast to this finding, Albillos et al. observed signs of cellular activation in peripheral blood monocytes of patients with decompensation of cirrhosis, indicated by increased number of CD14+ cells expressing HLA-DR and CD80 co-stimulatory molecules, and increased spontaneous and LPS-stimulated TNFα-expression [56]. These seemingly contradictory findings might be explained by the high heterogeneity of the patients, with inclusion of patients displaying more severe decompensations requiring ICU admissions in the former study. Taken together, these two studies illustrate that the immune phenotype changes dynamically with disease progression, and that cross-sectional studies are only able to capture a snapshot. 

More recent studies have further detailed that immunoparesis in ACLF is attributed to the expansion of MER receptor tyrosine kinase positive (MERTK+) monocytes (CD14+HLA-DR+MERTK+) [20], monocytic myeloid-derived suppressor cells (M-MDSCs) characterized by CD14+CD15-CD11b+HLA-DR- [21], and intermediate CD14++CD16+ monocytes [57]. MERTK+ monocytes have an impaired response to LPS stimulation and possibly contribute to secondary infections. Intermediate suppressive monocyte subsets are characterized by attenuated pro-inflammatory cytokine production in response to TLR2, TLR4 and TLR9 stimulation and higher production of anti-inflammatory/immunosuppressive cytokines such as IL-10 [20,21]. Functional alterations are also evident in classical CD14++CD16-monocytes of patients with ACLF. This subset of monocytes, which is considered to be highly phagocytic [58], exhibits reduced expression of TLR2 and TLR4 and more severely impaired phagocytic capacity and oxidative burst response compared to patients with AD cirrhosis [57]. The latter was found to be a result of diminished expression of interferon regulatory factor 8 (*IRF8*), a transcriptional activator of the oxidative burst response, which was only detected in ACLF but not in AD monocytes. In the same study, transcriptome analysis of the classical monocyte subset revealed upregulation of genes associated with immune dampening responses such as scavenger receptors (*CD163*, *MRC1*, *CD36*), suppressive cytokines (*IL-10*), chemokines (*CCL22*) as well as *MERTK*. Interestingly, the authors demonstrated that the phagocytic capacity of ACLF monocytes can be partially restored by targeting immunometabolism via inhibition of glutamine synthetase [57]. Another feature of monocytes from patients with AD cirrhosis is the downregulation of T-cell immunoglobulin domain and mucin domain-containing molecule-3 (Tim-3), most probably elicited by endotoxemia [59]. This finding was associated with a hypersensitive response to LPS challenge, decreased HLA-DR expression and reduced phagocytic activity. Alterations in monocyte function are clinically relevant, since persistently reduced HLA-DR expression as a surrogate marker of monocyte dysfunction was correlated with secondary infection and mortality [21,60].

### 6.2. Neutrophils

Neutrophils account for 55–70% of all circulating white blood cells and are among the first line of immune cells to be recruited to the site of infection. Whereas cirrhotic patients often present neutropenia [61], patients with ACLF have higher absolute neutrophil counts compared to healthy controls [62], probably induced by higher circulating granulocyte colony-stimulating factor (G-CSF) levels [4]. Neutrophils in patients with advanced cirrhosis display high levels of activation markers such as CD11b, a β_2_-integrin that mediates firm adhesion of neutrophils to cytokine-activated endothelium, and epidermal growth factor-like molecule containing mucin-like hormone receptor [63]. However, this is often combined with defects in their core functions, i.e., phagocytosis, respiratory burst and degranulation (exocytosis) [64]. These observations are suggestive of chronic intravascular activation of neutrophils, eventually leading to an exhausted phenotype.

In keeping with elevated molecules necessary for cell adhesion (e.g., VCAM-1, CD11b), and chemotactic signals such as IL-8 produced by immune cells, vascular endothelial cells and hepatocytes, neutrophils of patients with cirrhosis display increased adhesion to microvascular endothelium [65], but impaired ex vivo and in vivo transendothelial migration. The underlying mechanisms probably involve attenuated IL-33/ST2 signaling [66] as well as diminished levels of CD62L (L-selectin) due to activation-induced shedding [65].

It is also long-known that neutrophils from patients with alcoholic cirrhosis (active alcoholism is the second most frequent trigger of ACLF) display reduced phagocytic capacity towards Gram-positive and Gram-negative bacteria [67]. The reduced phagocytic capacity of neutrophils is independent of cirrhosis etiology [62]. In terms of ROS production, which is needed for respiratory burst, neutrophils of patients with ACLF produce higher basal levels of ROS, indicating a primed state of neutrophils [62]. On the other hand, ROS production upon fMet-Leu-Phe (fMLP)-stimulation in patients with alcoholic cirrhosis is reduced [68]. The diminished production of ROS was attributed to markedly reduced phospholipase C activity [69], deficient phosphorylation [64] as well as decreased baseline protein expression of components of the NADPH oxidase complex [68]. The latter could be explained by a defective mTOR-dependent translational machinery and degradation of gp 91phox/NOX2 through plasma elastase, which is present in high levels in plasma of patients with advanced cirrhosis. Together with defective myeloperoxidase exocytosis, which is possibly a consequence of decreased activation of AKT and p38-MAP-kinase, impaired ROS production contributes to deficient bactericidal activity [64,67]. 

Apart from impaired bacterial killing, CD11b+CD16+ neutrophils of patients with ACLF overexpress CXCR1 and CXCR2, the chemokine receptors recognizing IL-8, and induce hepatocyte death in vitro by direct contact and by release of inflammatory mediators. This finding provides another link between impaired neutrophil function and immunopathology [62], whereby dying hepatocytes release DAMPs which further activate the innate immune system. Neutrophil dysfunction is highly clinically relevant, as impaired respiratory burst and phagocytic activity correlate with higher risk of organ failure and mortality [70,71]. Elevated neutrophil-to-lymphocyte ratio, which can be easily calculated, predicts poor survival in patients with ACLF and proved to be higher in the ACLF than AD group [72].

### 6.3. Macrophages

Since ACLF is predominantly a systemic disease, information on immune cells such as macrophages residing in tissue and organs is scarce compared to their circulatory counterparts. This is mostly true for extrahepatic organs although there is some direct evidence on the role of macrophages in the liver. For instance, it has been reported that an expansion of hepatic macrophages expressing high levels of MERTK and CD163 occurs in ACLF patients undergoing liver transplantation [20,57]. These authors also provide evidence that MERTK+ macrophages accumulate in mesenteric lymph nodes, where these macrophages are phenotypically MERTKhigh/CD163high/HLA-DRhigh and functionally endotoxin tolerant. Furthermore, since soluble CD163, which is shed by activated macrophages, increases with severity of ACLF and predicts mortality, it was proposed that Kupffer cells, the liver resident macrophages which constitutively express high levels of scavenger receptors and IL-10, play an important role in the development and course of ACLF [73].

## 7. Mediators of Inflammation in ACLF

The dysfunctional innate immune system in ACLF patients is characterized by increased circulating levels of small proteins (cytokines, chemokines and growth factors) and lipids (bioactive lipid mediators) that signal for exuberant inflammatory and immune responses. 

### 7.1. Cytokines

Cytokines are low-molecular-weight glycoproteins that orchestrate the effectiveness of innate immunity by inducing local inflammation and systemic acute responses [74,75]. The production of cytokines by leukocytes is one of the initial steps of the inflammatory cascade. Once released, cytokines bind specific receptors in their target cells [75,76,77]. Although leukocytes are the major source of cytokines, parenchymal cells are increasingly recognized to also produce inflammatory cytokines and to interact with leukocytes to optimize immune responses [78]. Cytokines are also important in initiating, amplifying and mediating adaptive immunity [79]. Cytokines can be classified in different families including TNF, IL-6 families and interferon (α, β, and γ) families. Cytokines can also be categorized according to their role as pro-inflammatory or anti-inflammatory. TNF-α, IL-1β and IL-6 are well-characterized as pro-inflammatory cytokines, whereas IL-4, IL-10 and IL-1 receptor antagonist (IL-1ra) are considered anti-inflammatory [79]. The presence of increased circulating levels of TNFα and IL-6 in patients with cirrhosis without infections were described decades ago [80,81], but comprehensive characterization of these inflammatory mediators in AD cirrhosis and ACLF and their correlation with disease severity and organ failures were described more recently [4,5]. Circulating levels of cytokines in patients with ACLF are of similar magnitude to those reported in patients with sepsis. Therefore, the term “cytokine storm”, which defines the exacerbated production of these mediators as consequence of overactivated immune system accompanied by systemic inflammation, commonly observed in sepsis-like diseases [59] is also pertinent to ACLF.

IL-6 is a pleiotropic cytokine produced in response to infections and tissue injuries. IL-6 synthesis and secretion is induced upon stimulation of TLR4 by LPS, IL-1β or TNF-α and is one of the major stimuli for the release of hepatic acute phase proteins. IL-6 strongly correlates with the development of renal impairment and mortality in patients with cirrhosis and bacterial peritonitis [82]. The inhibition of IL-6 for the prevention or treatment of inflammatory diseases has been extensively approached [83]. For example, the neutralization of IL-6 signal by using the humanized anti-IL-6 receptor antibody tocilizumab, has been used in patients with systemic hyperinflammatory response including sepsis and macrophage activation syndrome in rheumatoid arthritis [84,85]. However, tocilizumab long-term trials have shown significant increments of transaminase levels requiring the need for regular monitorization and dose adjustments [86]. More recently, tocilizumab in combination with remdesivir, chloroquine and others has been repurposed for the treatment of the cytokine release syndrome in cirrhotic patients with Covid-19 [87]. 

### 7.2. Chemokines 

Chemokines consists of a superfamily of chemoattractant-related ligands and receptors that participate in the regulation of the immune system and inflammatory responses [88]. Based on the structural criteria, chemokines are classified into four subfamilies: CXC, CC, (X)C, and a single member of the CX3C subfamily (CX3CL1 or fractalkine). The pro-inflammatory chemokine CXCL8 (also known as IL-8) is produced by liver cells, including hepatocytes, stellate cells, and Kupffer cells and serves as predictor of mortality in ACLF [62]. Of interest, circulating CXCL10 levels have been shown to predict ACLF development and survival in cirrhotic patients with portal hypertension receiving TIPS [29]. 

### 7.3. Growth Factors 

Growth factors such as granulocyte-macrophage colony stimulating factor (GM-CSF) and G-CSF are implicated in hematopoiesis and proliferation of hepatic progenitor cells in liver failure [89]. Indeed, G-CSF has been considered for the treatment of ACLF with improvement of liver function and survival [90] (see below). The transforming growth factor β (TGF-β) is also associated with ACLF severity and survival and increased levels have been reported in non-survivor patients with hepatitis B infection [91].

### 7.4. Lipid Mediators

Lipid mediators are bioactive lipids generated from structural lipid species (i.e., phospholipids containing polyunsaturated fatty acids (PUFA)), which compose the lipid bilayer of cell membranes [92]. Most lipid mediators are derived from omega-6 and omega-3 PUFAs, which are released on demand in response to an inflammatory stimulus from cell membrane into the cytosol by phospholipase A_2_ [93,94]. In the cytosol, PUFA are readily converted by cyclooxygenase (COX), lipoxygenase (LOX) and cytochrome P450 (CYP) enzymatic pathways into an array of biologically active lipid mediators, which are released to exert their actions as autacoids [93,95]. The essential omega-6 PUFA arachidonic acid (AA) is the major substrate for the intracellular biosynthesis of eicosanoids. The eicosanoid family consists of PGs, thromboxane A_2_ (TXA_2_), LTs, lipoxins (LXs) and epoxyeicosatrienoic acids (EETs). With the exception of LXs, the majority of eicosanoids have pro-inflammatory properties [96] and in fact, PGs and TXA_2_ are the prime targets for non-steroidal anti-inflammatory drugs (NSAIDs) [97]. Similar to cytokines, eicosanoids are massively released by leukocytes in response to infections or tissue injury originating the so-called “eicosanoid storm” [92]. In contrast to AA, the omega-3 PUFAs eicosapentaenoic (EPA) and docosahexaenoic (DHA) acids are converted by the COX, LOX and CYP pathways into potent anti-inflammatory lipid mediators [98]. These mediators are generically known as “specialized pro-resolving mediators” or SPM (i.e., resolvins, protectins and maresins), which have attracted much attention in recent years because they do not only act as ‘braking signals’ of unremitting inflammation, but also play critical roles in the dynamic resolution of tissue inflammation [97]. 

**Figure 1:**
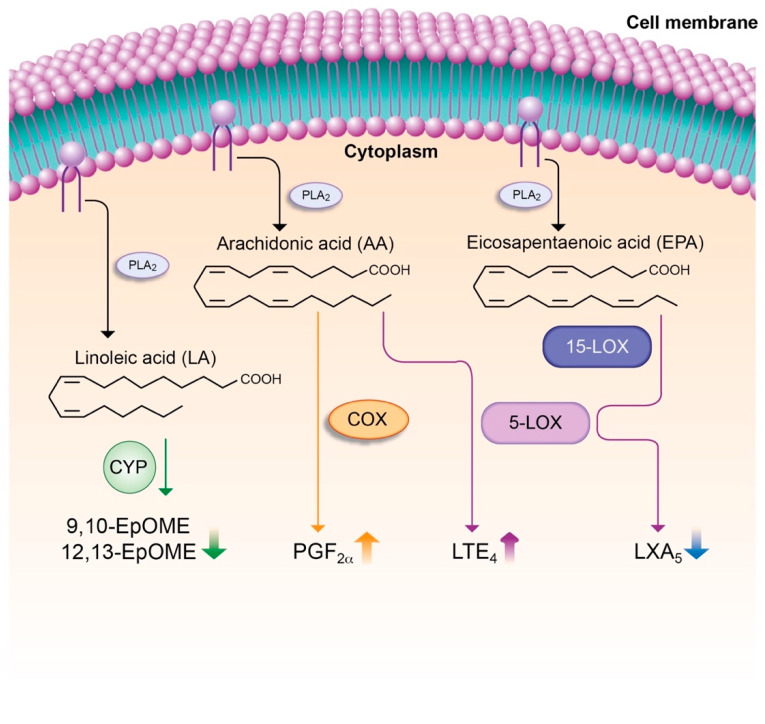
Imbalanced formation between pro-inflammatory and anti-inflammatory lipid mediators in leukocytes from patients with AD cirrhosis and ACLF. In response to inflammatory stimulus, omega-6 (linoleic acid (LA) and arachidonic acid (AA)) and omega-3 (eicosapentaenoic acid (EPA)) polyunsaturated fatty acids (PUFA) are released from cell membrane phospholipids into the cytosol by phospholipase A_2_ (PLA_2_). In the cytosol, unesterified free PUFA serve as available substrates for cyclooxygenase (COX), lipoxygenase (5-LOX/15-LOX) and cytochrome P450 (CYP) enzymatic pathways, which generate an array of bioactive lipid mediators. Patients with AD cirrhosis and ACLF present elevated levels of AA-derived pro-inflammatory and vasoconstrictor lipid mediators such as LTE_4_ and PGF_2α_, in parallel with decreased levels of the EPA-derived product LXA_5_, which is an anti-inflammatory and pro-resolving lipid mediator. In addition, the CYP-products derived from LA, 9,10-EpOME and 12,13-EpOME, which are produced by leukocytes during oxidative burst, were remarkably suppressed in ACLF patients, indicating poor bactericidal activity.

Little is known about the role of lipid mediators in ACLF. O’Brien et al. provided evidence that PGE_2_ drives immunosuppresion and increases the risk of infection in AD cirrhosis [22]. Subsequently, China et al. described that ACLF patients can be differentially categorized into two lipid mediator phenotypes (i.e., hyperactivated and hypoactivated) in their therapeutic response to human serum albumin (HSA) infusions [99]. The same authors reported that patient survival was associated with a shifted profile in the levels of SPMs [100]. More recently, our laboratory performed targeted analysis of 100 bioactive lipid mediators in more than 200 patients with AD cirrhosis with and without ACLF [101]. This study revealed elevated levels of pro-inflammatory and vasoconstrictor eicosanoids including LTE_4_ and PGF_2α_, in parallel with decreased levels of the pro-resolving SPM LXA_5_ in patients with ACLF [101] (Figure 1). In these patients, LTE_4_ levels were strongly correlated with IL-8 and the necrosis/apoptosis marker K18, whereas the pro-resolving LXA_5_ had a negative correlation with inflammation and cell death [101]. On the other hand, some lipid mediators derived from linoleic acid, 9(10)-epoxy-9Z-octadecenoic acid (EpOME) and 12(13)-EpOME, which are indicators of effective bactericidal activity, were remarkably suppressed in ACLF patients [101] (Figure 1). Together, these data suggest that systemic inflammation in ACLF can be driven by an imbalance formation between pro-inflammatory and anti-inflammatory/pro-resolving lipid mediators. 

## 8. Therapeutic Approaches to Limit Systemic Inflammation in ACLF

The most effective therapy for ACLF patients is liver transplantation. However, the availability of suitable organ donors is a major limitation. Therefore, finding a cure to prevent organ failures by limiting excessive systemic inflammation without inducing immunosuppression is an unmet need in patients with AD cirrhosis at risk of developing ACLF. At present, short- and long-term infusions of HSA are one of the few approved systemic therapies for the prevention of paracentesis-induced circulatory dysfunction and development of ascites and hepatorenal syndrome during an episode of spontaneous bacterial peritonitis (SBP) in patients with AD cirrhosis [102,103]. The therapeutic use of HSA in these patients was recently corroborated in a multicenter, randomized study, in which the long-term prophylactic administration of HSA was effective in reducing hospital readmissions and prolonging survival [104]. More recently, the pilot-PRECIOSA study aimed at identifying the optimal HSA dosage able to normalize HSA concentrations in patients with AD cirrhosis, and the randomized controlled INFECIR-2 study aimed at comparing the efficacy of adding HSA to standard medical therapy with antibiotics in patients with AD cirrhosis and active non-SBP bacterial infection, have demonstrated that both short- and long-term HSA treatments induce significant systemic immunomodulatory effects [105]. Specifically, HSA was shown to act as a disease-modifying anti-inflammatory agent with the ability to reduce the circulating levels of inflammatory cytokines in patients with AD cirrhosis [105]. Previously, O’Brien et al. had demonstrated that HSA was able to revert PGE_2_-induced immune dysfunction in patients with AD cirrhosis and ACLF [22]. This laboratory also demonstrated that plasma IL-4 levels served as a good marker of improvement in the degree of systemic inflammation after HSA treatment in these patients [100]. Other therapies based on HSA but alternative to infusions are extracorporeal liver support systems based on albumin dialysis or plasma exchange. This is the case of recent studies showing that patients treated with extracorporeal liver support systems had improved survival [106,107,108]. 

**Figure 2:**
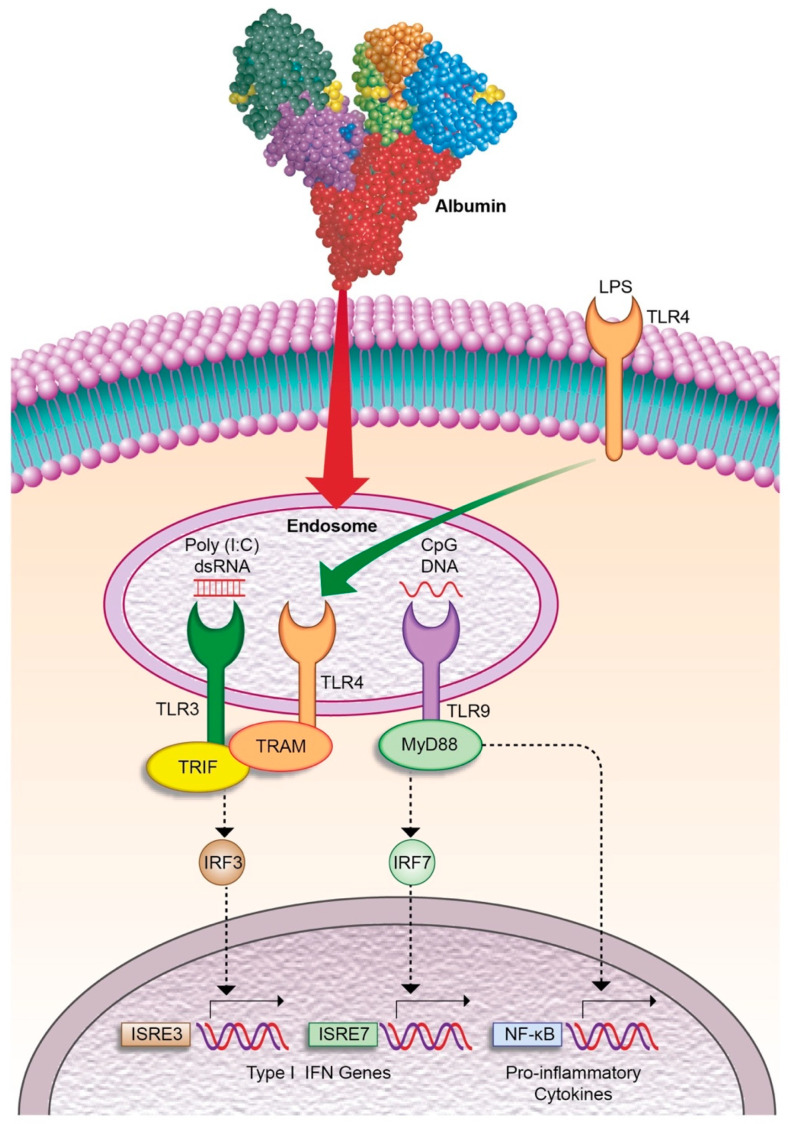
Human serum albumin (HSA) exerts immunomodulatory effects in leukocytes by blocking Toll-like receptor (TLR) signaling pathways in the endosomal compartment. HSA is internalized by leukocytes and in the endosomes inhibits inflammatory cytokine production induced by bacterial single-strand CpG-DNA, which binds to its cognate receptor TLR9 and triggers the signaling by recruitment of myeloid differentiation primary response gene 88 (MyD88). HSA also inhibits other endosomal TLRs such as TLR3, which is activated by double-strand RNA (i.e., poly (I:C)) and TLR4, which, after binding to LPS, translocates to the endosome. Both TRL3 and TLR4 signal via TIR-domain-containing adapter-inducing interferon-β (TRIF), which mediates type I interferon (IFN) responses.

Although traditionally the therapeutic effects of HSA in patients with AD cirrhosis were attributed to its oncotic, antioxidant and scavenging properties [103], new mechanisms have been recently described that contribute to understand the immunomodulatory and anti-inflammatory properties of the albumin molecule. In particular, ex vivo experiments in isolated leukocytes from patients with AD and ACLF have provided evidence that HSA abolish cytokine expression and release induced by bacterial DNA rich in unmethylated CpG-DNA [109]. These anti-inflammatory effects of HSA were independent of its oncotic and scavenging properties and were reproduced by incubating the leukocytes with recombinant human albumin. In addition, HSA exerted widespread changes on the leukocyte transcriptome, specifically in genes related to the endosomal compartment involved in cytosolic DNA sensing and type I interferon responses. Consistent with this, flow cytometry and confocal microscopy analyses revealed that HSA was taken up by leukocytes and internalized in endosomes, the compartment where CpG-DNA binds to TLR9, its cognate receptor (Figure 2). In this compartment, HSA also inhibited poly(I:C)- and LPS-induced interferon regulatory factor 3 (IRF3) phosphorylation and TIR-domain-containing adapter-inducing interferon-β (TRIF)-mediated responses, which are exclusive of endosomal TLR3 and TLR4 signaling, respectively (Figure 2). Importantly, the immunomodulatory actions of HSA did not compromise leukocyte defensive mechanisms such as phagocytosis, efferocytosis and intracellular ROS production, and thus appear to not induce immunosuppression. Together, these findings indicate that similar to that reported for other cell types such as hepatocytes and endothelial cells, albumin internalizes in leukocytes and modulates the responses to PAMPs through interaction with endosomal TLR signaling pathways [109].

Other therapies currently explored to treat excessive systemic inflammation and restore the immunological response in patients with AD cirrhosis and ACLF are G-CSF, TLR4 antagonists, IL-22 and stem cell therapy. Therapy with G-CSF, which mobilizes bone marrow stem cells and impacts on hepatocyte proliferation as it also acts as a growth factor, has been tested in patients with ACLF in four randomized clinical trials [110,111,112] including the GRAFT study, which was stopped after interim analysis because lack of additional effects to standard medical treatment [113]. In any case, administration of G-CSF subcutaneously was shown to be safe in patients with ACLF, to increase the number of CD34+ cells and to promote hepatic regeneration. Additionally, a recent study has demonstrated that autologous infusion of G-CSF-induced CD34+ cells effectively improves liver function and HSA levels up to one year although this benefit was not sustained at the long-term [114]. Moreover, G-CSF treatment has been shown to prevent hepatorenal syndrome, hepatic encephalopathy and sepsis [110,112]. Another therapeutic target in ACLF is TLR4, which plays an essential role in mediating organ injury in this condition. Recently, TAK-242, TLR4 antagonist was experimentally tested in vivo in different rodent models of ACLF and in vitro in monocyte and hepatocyte cell lines. The results obtained indicated that in addition to reduce cytokine levels and hepatocyte cell death, TAK-242 was able to increase survival in mice experimentally induced to ACLF [115]. A recent study in a mouse model mimicking the key features of ACLF has provided evidence that IL-22 treatment reprograms impaired regenerative pathways and protects against bacterial infection [116], although significant discrepancies between the data reported by these authors and data reported earlier in human ACLF have been raised [24]. Finally, stem cell therapy has been tested in two open-label controlled studies in patients with ACLF, who received umbilical cord-derived mesenchymal stem cells (MSC) [117] or allogenic bone marrow-derived MSC [118]. In these studies, MSC infusions increased survival rate, improved liver function and increased serum albumin, although none of them addressed the effects on systemic inflammation.

## 9. Conclusions

The ACLF syndrome represents a new paradigm among the diseases characterized by the presence of an excessive systemic inflammatory response leading to organ failure(s). The ACLF syndrome thus is a suitable disease condition to investigate the mechanisms underlying systemic inflammation and tissue damage. At present, little is known about the individual participation of each immune cell type to this process, but ongoing studies using cutting edge technologies such as single blood cell-RNA seq will likely provide new insights soon. The relative contribution of the innate and adaptive immune systems to systemic inflammation and immunopathology in patients with AD cirrhosis evolving to ACLF also needs to be elucidated. Future studies are also needed for the identification of the triggers of the systemic inflammatory response that leads to end-organ dysfunction in the context of ACLF. Finally, it is of utmost importance to find an appropriate intervention that would reduce inflammation without inducing immunosuppression to these patients.
